# Factors associated with nurses’ adherence to standard precautions: implications for healthcare-associated infection prevention and occupational safety in Taiwan

**DOI:** 10.3389/fpubh.2026.1896298

**Published:** 2026-08-10

**Authors:** Aih-Fung Chiu, Chun-Fung Chiu, Chun-Man Hsieh

**Affiliations:** 1 Department of Nursing, Cheng Shiu University, Kaohsiung, Taiwan; 2 Kaohsiung Veterans General Hospital, Kaohsiung, Taiwan; 3 Department of Nursing, Tajen University, Yanpu Township, Taiwan

**Keywords:** adherence, healthcare-associated infections, infection prevention, nurses, occupational safety, standard precautions

## Abstract

**Background:**

Healthcare-associated infections (HAIs) remain a major threat to patient safety and healthcare workforce protection, particularly after the COVID-19 pandemic. Standard precautions (SPs) are the most fundamental infection prevention measures used to reduce pathogen transmission and occupational exposure in clinical settings. Although awareness of SPs has increased, adherence among nurses continues to vary across practice domains. Factors such as familiarity with SP-related knowledge, years of nursing experience, and perceived barriers in daily practice may influence compliance; however, related evidence among hospital nurses in Taiwan is still limited.

**Materials and methods:**

This cross-sectional study recruited nurses who were directly involved in patient care at a teaching hospital in southern Taiwan using convenience sampling. Nurse managers without direct patient care responsibilities were excluded. Adherence to standard precautions (SPs) was assessed using the Chinese version of the Compliance with Standard Precautions Scale (CSPS). Data regarding perceived barriers, self-rated familiarity with SP-related knowledge, and personal characteristics were also collected. Multiple linear regression analysis was performed to identify factors associated with adherence to SPs.

**Results:**

Participants had a mean age of 37.7 years; most were female (96.2%) and held a bachelor’s degree or above (71.2%). The mean self-rated familiarity score with SP-related knowledge was 7.8 ± 1.5 on a 10-point scale. The average adherence rate across the five CSPS domains was 78.6%, and the overall item-level adherence rate was 78.9%. Familiarity with SP-related knowledge showed the strongest positive association with SP adherence, while years of nursing experience and perceived barriers were negatively associated with compliance (all *p* < 0.05). Among the five domains, sharps disposal showed the lowest adherence.

**Conclusion:**

Although nurses demonstrated generally moderate to high adherence to SPs, deficiencies remained in high-risk practices, especially sharps disposal. Strengthening familiarity with SP-related knowledge and reducing workplace barriers may improve occupational safety, lower the risk of HAIs, and support more sustainable infection prevention strategies at both institutional and public health levels.

## 1 Introduction

In recent decades, emerging infectious diseases, such as COVID-19, and other biological hazards have highlighted the risks of healthcare-associated infections (HAIs). HAIs may occur throughout the patient care process and not only compromise patient safety—resulting in increased morbidity and mortality, prolonged hospitalization, disability, and additional financial burdens—but also increase occupational exposure risks for healthcare workers (1, 2). As healthcare systems continue to face challenges related to infectious disease outbreaks and workforce shortages, infection prevention has become not only a clinical priority but also a critical component of public health preparedness and health system resilience (1, 2). In addition, HAIs increase healthcare costs, prolong hospital stays, and place substantial pressure on healthcare systems, making infection prevention an important issue for both patient safety and public health management (1).

Standard precautions (SPs) are the primary infection prevention strategy designed to reduce the risk of pathogen transmission and prevent the spread of infections in healthcare settings (3). They represent the minimum infection prevention practices required to achieve a basic level of infection control (1). The essential components of SPs include hand hygiene, personal protective equipment (PPE), safe injection practices, environmental cleaning, and waste management (1, 3, 4). As frontline healthcare providers, nurses are among the most critical professionals in patient care and provide care regardless of patients’ infection status (4, 5). Therefore, nurses are at particularly high risk of occupational exposure and infection. Consistent adherence to SPs is recognized as one of the most effective strategies for reducing HAIs, preventing occupational exposure, and maintaining a safe healthcare environment for both patients and healthcare workers (2).

However, nurses’ adherence to SPs remains variable, and suboptimal compliance has been widely documented. A study conducted during the COVID-19 period reported an overall SP compliance rate of 76.0% among clinical nurses, with significant gaps persisting in waste and sharps disposal (6). Another recent multicenter study further confirmed that nurses’ compliance with SPs remained suboptimal during the COVID-19 period, although some improvement was observed compared with pre-pandemic levels (7). These findings suggest that adherence to SPs continues to vary across domains, particularly in high-risk practices such as sharps disposal and waste management. This also indicates that improving awareness alone may not be sufficient to ensure sustained compliance, and both individual and organizational factors should be considered simultaneously (7, 8). Taiwan has long emphasized hospital infection control, particularly during the COVID-19 period. Therefore, understanding nurses’ adherence behaviors and perceptions regarding the implementation of SPs is essential for developing effective infection prevention strategies.

Despite these ongoing concerns, previous studies have shown that adherence may be associated with multiple factors, including self-rated familiarity with SP-related knowledge, years of nursing experience, and perceived barriers in clinical practice. In particular, domain-specific variations in adherence and the influence of perceived barriers on compliance behaviors remain insufficiently explored during and beyond the COVID-19 pandemic. Regarding the perceived barriers to adherence to SPs, previous literature has shown that barriers such as time constraints, emergency situations, limited availability of protective equipment, discomfort associated with PPE use, and the perceived inconvenience of guideline implementation may reduce nurses’ willingness to comply with SPs (7, 9). These perceived barriers may directly influence nurses’ compliance behaviors and are considered important modifiable factors in infection prevention practice. These findings highlight the importance of developing targeted strategies and workplace policies to improve adherence.

This issue has become even more important during and beyond the COVID-19 pandemic, when healthcare systems are expected to maintain long-term infection prevention capacity beyond emergency outbreak responses. Thus, this study aimed to examine nurses’ adherence to SPs and associated factors among hospital nurses in Taiwan during the COVID-19 period, with particular attention to domain-specific variations, self-rated familiarity with SP-related knowledge, and perceived barriers to implementation.

## 2 Methods

### 2.1 Design

A cross-sectional study using convenience sampling was conducted among nurses working at a teaching hospital in southern Taiwan during a period of heightened infection prevention and control demands associated with the COVID-19 pandemic. Data were collected between January 10 and April 30, 2021, using a structured self-administered questionnaire. Eligible participants were nurses directly involved in patient care. Data collection was conducted in designated unit meeting rooms considered suitable for questionnaire administration. Before participation, the researchers explained the study purpose and procedures to the participants. Participants completed the questionnaires independently, and research staff were available on-site to provide clarification when necessary. Participation was voluntary and anonymous to ensure confidentiality and protect participants’ rights.

### 2.2 Participants

The study was conducted among nurses working at a teaching hospital in southern Taiwan. Nurses who were directly involved in patient care (frontline nurses) were eligible for inclusion, whereas head nurses and managerial staff who did not provide direct patient care were excluded. A total of 332 nurses were approached, and 264 returned valid self-administered questionnaires, yielding a response rate of 79.5%.

### 2.3 Measures

Measures included participants’ demographic and work-related characteristics, adherence to SPs, familiarity with SP-related knowledge, SP-related training experience, and perceived barriers to SP adherence. Details are provided below.

#### 2.3.1 Demographic and work-related characteristics

Participants’ demographic and work-related characteristics were collected using a structured self-administered questionnaire. These variables included gender, age, educational attainment (vocational nursing school, junior college, university, graduate school, or above), job title (nurse or nurse practitioner), work unit (emergency, critical care, or special units vs. general wards and outpatient clinics), years of nursing experience, hours of SP-related courses completed in the past year, and self-rated familiarity with SP-related knowledge, which was measured using a 0–10 numerical rating scale.

#### 2.3.2 Compliance with standard precautions scale (CSPS)

Adherence to standard precautions (SPs) was treated as the dependent variable in this study and was assessed using the Chinese version of the Compliance with Standard Precautions Scale (CSPS), which was developed by Lam in 2011 (4). The 20-item scale evaluates SP practices across five domains: use of protective devices, disposal of sharp instruments, disposal of waste, decontamination of spills and used articles, and prevention of person-to-person cross-infection. Items are rated on a 4-point Likert scale (0 = “never” to 3 = “always”), with total scores ranging from 0 to 60. Consistent with the original scoring method, item-level adherence rates were calculated as the percentage of participants responding “always,” whereas reverse scoring was applied to negatively worded items. The average adherence rate for each domain was calculated by dividing the sum of item-level adherence rates by the number of items in that domain. The CSPS has demonstrated good reliability and validity, with test–retest reliability ranging from 0.73 to 0.79 and criterion validity of 0.76 (4). In the present study, Cronbach’s alpha was 0.642. This value was slightly lower than that reported in the original validation study by Lam (4). One possible explanation was that the study sample was heterogeneous, as nurses were recruited from different clinical units, and significant differences in adherence to standard precautions were observed across these units.

#### 2.3.3 Perceived barriers to SP adherence

Perceived barriers to SP adherence were treated as an independent variable in this study, and the scale was developed by the researchers based on a review of the literature and clinical experience. The items were reviewed and revised to ensure face validity. The perceived barriers scale comprised eight items, originally rated on a 5-point Likert scale ranging from 1 (strongly disagree) to 5 (strongly agree). For statistical analysis and internal consistency estimation, item responses were recoded to a 0–4 scale, with higher total scores indicating greater perceived barriers. Construct validity was examined using exploratory factor analysis with principal component analysis and varimax rotation; factor loadings exceeding 0.40 were considered acceptable. The analysis yielded a single-factor structure, which was identified as “perceived barriers” (see Supplementary Table S1). The scale demonstrated high internal consistency, with a Cronbach’s alpha coefficient of 0.844, exceeding the commonly accepted threshold of 0.80 (10).

### 2.4 Ethical considerations

The study protocol was reviewed and approved by the Institutional Review Board of Kaohsiung Armed Forces General Hospital (KAFGHIRB No. 110–039). All participants provided written informed consent before participation.

### 2.5 Statistical analysis

Data were analyzed using SPSS version 23.0 (SPSS Inc., Chicago, IL, USA). Descriptive statistics are presented as means ± standard deviations (*SD*) or frequencies and percentages. Differences in adherence to SPs among categorical groups were compared using Student’s t tests or one-way analysis of variance (ANOVA). Pearson’s correlation analysis, followed by forward stepwise multiple linear regression analysis, was performed to identify significant factors associated with adherence to SPs. Only variables with significant results in the univariate analysis were entered into the forward stepwise multiple linear regression analysis, and only variables retained through the stepwise selection procedure were included in the final model. Statistical significance was defined as a two-tailed *p* < 0.05.

## 3 Results

### 3.1 Participant characteristics

Table 1 presents the demographic and work-related characteristics of the participants. The study included 264 nurses, comprising 254 women and 10 men, with a mean age of 37.7 ± 10.1 years (range: 21–64 years). Most participants were clinical nurses (89.8%), and 71.2% had attained a university degree or higher. Approximately 54.2% of participants worked in special units, including emergency departments, intensive care units, negative-pressure wards, operating rooms, and respiratory care wards. The mean length of nursing experience was 15.1 ± 10.3 years, ranging from 0.3 to 42.5 years. Participants reported receiving an average of 8.0 ± 1.6 h of SP-related courses in the past year (range: 4–10 h), and the mean self-rated familiarity with SP-related knowledge was 7.8 ± 1.5 on a 0–10 numerical rating scale.

**Table 1:** Participants’ characteristics among nurses (*N* = 264).

Variables	n	(%)	*Mean ± SD*
Gender			
Women	254	(96.2)	
Men	10	(3.8)	
Age (years)			37.7 ± 10.1
<35	110	(41.7)	
≥35–<45	76	(28.8)	
≥45	78	(29.5)	
Job title			
Nurse practitioners	27	(10.2)	
Nurse	237	(89.8)	
Education attainment			
Associate degree	76	(28.8)	
Bachelor’s degree	177	(67.0)	
Master’s degree	11	(4.2)	
Work units			
General ward or outpatient department	121	(45.8)	
Emergency, intensive care, or other special units ^a^	143	(54.2)	
Years of nursing experience			15.1 ± 10.3
<3 years	31	(11.7)	
3–<10 years	69	(26.1)	
10–<20 years	64	(24.2)	
≥20 years	100	(37.9)	
SPs-related courses in the last year (hours, range 4–10)			8.0 ± 1.6
Familiarity with SP-related knowledge (on a 0–10 numerical scale)			7.8 ± 1.5
Perceived barriers (8 items, potential range 8–40)			26.2 ± 6.8

SPs, Standard Precautions. ^a^ special units: negative pressure ward, surgery room, or respiratory care ward.

### 3.2 Perceived barriers to SP adherence

As shown in Table 1, the mean total score for perceived barriers to adherence to SPs was 26.2 ± 6.8 (possible range: 8–40), indicating a moderate level of perceived barriers.

### 3.3 Adherence to SPs (CSPS results)

Table 2 presents the individual adherence rates for each CSPS item, defined as the proportion of nurses reporting adherence to SPs. For negatively worded (reverse-scored) items, the response indicating correct practice was used to calculate the adherence rate. The individual adherence rates to SPs ranged from 18.2 to 99.6%. Across the five CSPS domains, adherence rates ranged from 50.5% in the “Disposal of sharps” domain to 90.9% in the “Decontamination of spills and used articles” domain. The overall average adherence rate across the five CSPS domains was 78.6%. Items with individual adherence rates below 50% included “The sharps box is disposed only when it is full” (item 6) at 18.2%, “I recap used needles after giving an injection” (item 4) at 35.6%, “I reuse a surgical mask or disposable personal protective equipment” (item 15) at 43.6%, and “I only use water for hand washing” (item 2) at 40.9% (Table 2).

**Table 2:** The adherence to the CSPS items and domains among nurses (*N* = 264).

Domains, items	Never	Rarely	Sometimes	Always	Adherence rate
Prevention of cross-infection (7 items)					81.2%
1. I wash my hands between patient contacts.	1 (0.4)	1 (0.4)	9 (3.4)	253 (95.8)	95.8%
2. I only use water for hand washing. ^§^	108 (40.9)	101 (38.3)	36 (13.6)	19 (7.2)	40.9%
3. I use alcoholic hand rubs as an alternative if my hands are not visibly soiled.	7 (2.7)	30 (11.4)	73 (27.7)	154 (58.3)	58.3%
8. I take a shower in case of extensive splashing even after I have put on Personal Protective Equipment.	7 (2.7)	12 (4.5)	11 (4.2)	234 (88.6)	88.6%
9. I cover my wound(s) or lesion(s) with waterproof dressing before patient contacts.	0 (0.0)	2 (0.8)	15 (5.7)	247 (93.6)	93.6%
11. I change gloves between patient contacts.	2 (0.8)	1 (0.4)	10 (3.8)	251 (95.1)	95.1%
12. I decontaminate my hands immediately after removal of gloves.	0 (0.0)	0 (0.0)	10 (3.8)	254 (96.2)	96.2%
Disposal of sharps (3 items)					50.5%
4. I recap used needles after giving an injection. ^§^	94 (35.6)	9 7(36.7)	44 (16.7)	29 (11.0)	35.6%
5. I put used sharp articles into sharps boxes.	4 (1.5)	2 (0.8)	0 (0.0)	258 (97.7)	97.7%
6. The sharps box is disposed only when it is full. ^§^	48 (18.2)	74 (28.0)	50 (18.9)	92 (34.8)	18.2%
Disposal of waste (1 items)					87.5%
17. Waste contaminated with blood, body fluids, secretion and excretion is placed in red plastic bags irrespective of the patient’s infection status	7 (2.7)	9 (3.4)	17 (6.4)	231 (87.5)	87.5%
Decontamination of spills and used articles (3 items)					90.9%
18. I decontaminate surfaces and equipment after use.	0 (0.0)	6 (2.3)	55 (20.8)	203 (76.9)	76.9%
19. I wear gloves to decontaminate used equipment with visible soils.	0 (0.0)	0 (0.0)	8 (3.0)	256 (97.0)	97.0%
20. I clean up spillage of blood or other body fluids immediately with disinfectants.	0 (0.0)	0 (0.0)	3 (1.1)	261 (98.9)	98.9%
Use of protective device (6 items)					82.9%
7. I remove Personal Protective Equipment (PPE) in a designated area.	0 (0.0)	3 (1.1)	18 (6.8)	243 (92.0)	92.0%
10. I wear gloves when I am exposed to body fluids, blood products, and any excretion of patients.	1 (0.4)	0 (0.0)	0 (0.0)	263 (99.6)	99.6%
13. I wear a surgical mask alone or in combination with goggles, face shield and apron whenever there is a possibility of a splash or splatter.	1 (0.4)	14 (5.3)	32 (12.1)	217 (82.2)	82.2%
14. My mouth and nose are covered when I wear a mask.	0 (0.0)	0 (0.0)	3 (1.1)	261 (98.9)	98.9%
15. I reuse a surgical mask or disposable Personal Protective Equipment. §	115 (43.6)	64 (24.2)	25 (9.5)	60 (22.7)	43.6%
16. I wear a gown or apron when exposed to blood, body fluids or any patient excretions.	1 (0.4)	13 (4.9)	36 (13.6)	214 (81.1)	81.1%
Average adherence for the five domains of CSPS	78.6%				
Average adherence for all CSPS items	78.9%				

CSPS, Compliance with Standard Precautions Scale. ^§^ indicates a reverse-scored question, and scoring was done using a reverse scoring method.

### 3.4 Factors associated with adherence to SPs

Table 3 presents the results of the univariate analyses comparing CSPS scores across categories of selected variables. Nurses working in general wards or outpatient departments had significantly higher CSPS scores than those working in emergency, intensive care, or other special units (*p* = 0.006). In addition, significant differences in CSPS scores were observed across categories of years of nursing experience (*p* = 0.001), with higher adherence observed among nurses with fewer years of experience. No significant differences in CSPS scores were found with respect to sex, job title, or educational attainment.

**Table 3:** Adherence to SPs by categories of selected variables (*N* = 264).

Variables	CSPS scores			
	*Mean*	*± SD*	*t/F*	*p*-value
Sex			0.180	0.857
Women (*n* = 254)	53.09	± 3.29		
Men (*n* = 10)	52.9	± 4.58		
Job title			0.143	0.886
Nurse practitioners (*n* = 27)	53.10	± 3.41		
Nurse (*n* = 237)	53.0	± 2.69		
Education attainment			1.306	0.273
Associate degree (*n* = 76)	52.5	± 2.91		
Bachelor’s degree (*n* = 177)	53.30	± 3.55		
Master’s degree (*n* = 11)	53.27	± 2.00		
Work units			2.791	0.006*
General ward/outpatient department (*n* = 121)	53.70	± 3.29		
Emergency, intensive care, or other special units (*n* = 143)	52.57	± 3.30		
Years of nursing experience				0.001*
<3 years (*n* = 31)	54.87	± 3.08		
3–<10 years (*n* = 69)	53.57	± 3.64		
10–<20 years (*n* = 64)	53.08	± 3.55		
≥20 years (*n* = 100)	52.21	± 2.76		

*p < 0.05; CSPS, Compliance with Standard Precautions Scale; SPs, Standard Precautions; Student *t*-tests or one-way analysis of variance with Scheffe’s test were used.

Table 4 shows the results of Pearson’s correlation analyses between continuous variables and CSPS scores. CSPS scores were positively correlated with the number of SP-related courses received in the past year and self-rated familiarity with SP-related knowledge, whereas they were negatively correlated with age, years of nursing experience, and perceived barriers to SP adherence (all *p* < 0.05).

**Table 4:** Correlation analyses between continuous variables and CSPS scores (*N* = 264).

Variables	Pearson’s *r*	*p*-value
Age	−0.187	0.002*
Years of nursing experience	−0.202	0.001*
SPs-related courses in the last year (hours, range 4–10)	0.144	0.020*
Familiarity with SPs (on a 0–10 numerical scale)	0.266	<0.001*
Perceptual barriers (8 items)	−0.244	<0.001*

*p < 0.05; CSPS, Compliance with Standard Precautions Scale; SPs, Standard Precautions; Pearson’s correlation analyses were used.

Variables significantly associated with CSPS scores in the univariate analyses were subsequently entered into the multiple linear regression model. Table 5 presents the results of the forward stepwise multiple linear regression analysis. Using the forward selection procedure, familiarity with SP-related knowledge was retained in Model 1, followed by years of nursing experience in Model 2 and perceived barriers to SP adherence in Model 3. The final model explained 13.9% of the variance in CSPS scores (Adjusted R² = 0.139).

**Table 5:** The independent factors of CSPS scores (*N* = 264).

Variables entered	Model 1	Model 2	Model 3
Level of familiarity with SPs^a^ (on a 0–10 numerical scale)			
β	0.266	0.278	0.241
B	0.603	0.630	0.546
B 95 % CI	(0.337 ∼ 0.869)	(0.370 ∼ 0.890)	(0.285 ∼ 0.808)
*p*-value	<0.001*	<0.001*	<0.001*
Years of nursing experience			
β		–0.217	–0.202
B		–0.071	–0.066
B 95 % CI		(–0.108 ∼ –0.033)	(–0.102 ∼ –0.029)
*p*-value		<0.001	0.001*
Perceptual barriers			
β			–0.180
B			–0.088
B 95 % CI			(–1.44 ∼ –0.032)
*p* -value			0.002*
R	0.266	0.343	0.386
R^2^	0.071	0.118	0.149
Adjust R^2^	0.067	0.111	0.139
R^2^ change	0.071	0.047	0.031
F change (Degrees of Freedom)	19.938 (1,262)	13.952 (1,261)	9.440 (1,260)
Sig. F change	<0.001*	<0.001*	0.002*

*p < 0.05; CSPS, Compliance with Standard Precautions Scale; Multiple linear regression analyses with the forward stepwise method were performed.

a On a 0–10 numerical scale, the higher the score, the higher the familiarity; The total adjusted explained variance was 13.9%.

## 4 Discussion

This study aimed to examine nurses’ adherence to SPs and the factors associated with their implementation. Using the CSPS, we observed a moderate-to-high level of adherence to SPs among the study sample; however, substantial variation across individual SP items was identified (Table 2), particularly in practices involving sharps handling, hand hygiene, and the use of personal protective equipment. These findings suggest that although overall adherence to SPs was relatively favorable, important gaps in infection prevention practices remain. Multiple linear regression analysis revealed that greater self-rated familiarity with SP-related knowledge was associated with higher adherence to SPs. This finding is consistent with previous studies showing that greater knowledge and familiarity with SPs are associated with better compliance among healthcare workers and nursing students (11, 12). However, not all studies have reported similar findings. A recent study of intensive care unit nurses found that knowledge of standard precautions was not significantly associated with performance, whereas health beliefs regarding standard precautions emerged as a significant predictor of compliance (13). In contrast, longer nursing experience and greater perceived barriers were associated with lower adherence to SPs (all *p* < 0.05; Table 5).

Our study revealed that the overall average adherence rate across the five CSPS domains was 78.6%, whereas the overall item-level adherence rate across all CSPS items was 78.9%. Our findings are comparable to recent evidence from studies conducted during and after the COVID-19 pandemic indicating moderate overall adherence to SPs, with persistent variability across domains, particularly in high-risk practices (6, 7). Unlike the low adherence rates reported in previous studies conducted prior to the COVID-19 pandemic (14, 15), our study revealed higher adherence rates during the pandemic, suggesting that heightened infection prevention awareness during the COVID-19 pandemic may have contributed to improved adherence to SPs.

Consistent with recent evidence from studies conducted during and after the COVID-19 pandemic, adherence to SPs related to sharps disposal remains suboptimal among practicing nurses, indicating that unsafe sharps-related behaviors continue to represent a critical gap in infection prevention practices (6, 7). In addition, two recent surveys using different scales to assess SPs also reported inadequate adherence among nursing professionals to practices related to unsafe sharps management (8, 16), hand hygiene, and the use of PPE (16). These findings underscore the need for targeted efforts to improve adherence to SPs. Nurses’ non-adherence to SPs may place them at risk of acquiring healthcare-associated infections (HAIs). These findings highlight the need to address specific areas of non-adherence to improve infection prevention practices and ensure the safety of both nurses and patients. Further research aimed at developing targeted interventions or strategies to improve adherence may also be warranted.

Regarding factors associated with adherence to SPs, high self-rated familiarity with SP-related knowledge was, as expected, a crucial predictor of adherence. Although a recent study reported no association between knowledge of and adherence to SPs (17), our findings are consistent with numerous other studies highlighting the importance of SP-related knowledge and education in improving adherence (2, 15, 18). Notably, in Taiwan, infection prevention and SP-related continuing education programs are routinely incorporated into hospital accreditation and staff training requirements. In our sample, nurses attended an average of 8.0 ± 1.6 h (range: 4–10) of infection control or SP-related courses per year and scored 7.8 ± 1.5 on a 0–10 numerical rating scale for self-rated familiarity with SP-related knowledge. This may partly explain the relatively high adherence to SPs observed in our study sample. Furthermore, self-rated familiarity with SP-related knowledge was significantly associated with adherence to SPs; therefore, hospital administrators and nursing supervisors may emphasize the importance of continuing education and regularly provide relevant training to support nurses’ adherence to SPs.

The second variable included in the regression model was years of nursing experience. The results indicated that nurses with more years of nursing experience tended to have lower scores on the CSPS. This finding was consistent with those of two previous studies. One study reported that nurses with more than 5 years of work experience had poorer adherence to SPs (19). One possible explanation is that adherence to routine infection prevention practices may decline over time as clinical tasks become increasingly familiar and habitual. As nurses gain more clinical experience, they may rely more on their own clinical judgment and previous experience when performing familiar procedures, rather than consistently following every component of standard precautions. This may contribute to lower adherence to SPs in routine clinical practice. Recent research has further highlighted that, rather than years of experience alone, patient safety climate and nurses’ individual perceptions of workplace safety may play a more critical role in shaping adherence to SPs (20, 21). A recent study conducted in critical care units showed that greater critical care experience was associated with higher compliance with infection prevention and control practices, suggesting that the relationship between professional experience and adherence may vary across healthcare settings and organizational contexts (22). Taken together, these findings suggest that nursing experience alone may not ensure consistent adherence to SPs. Regardless of years of clinical experience, continuous reinforcement of standard precautions and regular infection prevention education remain essential to promote sustained adherence among nurses.

In contrast, two other studies reported different findings. One study showed that years of experience were significantly and positively associated with adherence to SPs among 266 nurses in northern Jordan (15). Another study conducted in Brazil reported that nursing professionals (including nurses, nursing technicians, and assistants) with longer institutional experience (≥15 years) were more likely to adhere to SPs (16). Given these inconsistent findings, differences in healthcare settings, participant characteristics, and institutional safety culture may partly explain the variation across studies. Further research is needed to clarify the relationship between years of nursing experience and adherence to SPs. In the meantime, targeted educational and organizational interventions aimed at improving adherence to SPs may be beneficial regardless of nurses’ experience levels.

Perceived barriers were independently associated with adherence to SPs. This finding was consistent with those of two previous studies. Specifically, perceived barriers were strong predictors of adherence to SPs among 116 nurses (14) and 678 nursing students (11). In clinical practice, healthcare providers, including nurses, may choose whether to comply with SPs based on their perceptions. Previous studies have identified multiple barriers to adherence to SPs, including heavy workloads, inadequate resources, insufficient equipment, and organizational factors (23, 24). In the present study, perceived barriers encompassed a broader range of perceived difficulties, including both psychological factors (e.g., anxiety and exhaustion) and workplace-related challenges. These multidimensional difficulties may reduce healthcare workers’ capacity or willingness to consistently adhere to SPs in clinical practice. Therefore, interventions such as educational programs and organizational support should address both workplace conditions and healthcare workers’ psychological barriers to improve adherence to SPs.

### 4.1 Study limitations

Despite these important findings, several limitations of this study should be acknowledged. First, the cross-sectional design limits interpretation, and causal inferences cannot be drawn. The observed associations should therefore be interpreted with caution, and future studies employing experimental or longitudinal designs are needed to establish cause-and-effect relationships. Second, this study focused solely on nurses working in a teaching hospital in southern Taiwan. The use of convenience sampling may have introduced selection bias and limited the external validity of the findings. Future research involving larger and more diverse populations, as well as the use of random sampling techniques, is recommended to enhance generalizability. In addition, SP-related knowledge was assessed using a self-rated measure, which may not fully reflect participants’ actual knowledge and may therefore be subject to reporting bias. Future studies are encouraged to incorporate objective knowledge assessments to provide a more comprehensive evaluation of the relationship between SP-related knowledge and adherence to standard precautions. Furthermore, adherence to standard precautions was assessed using a self-administered questionnaire. Although anonymous participation and confidentiality were emphasized to minimize potential reporting bias, self-reported adherence may still be subject to reporting bias or social desirability bias and may not fully reflect actual clinical practice. Future studies incorporating direct observational methods or mixed assessment approaches are recommended to provide a more comprehensive evaluation of adherence to standard precautions. Finally, the multiple linear regression model explained only 13.9% of the variance in CSPS scores. This relatively modest explanatory power suggests that additional organizational and workplace-related factors, particularly patient safety climate and psychological safety, may contribute to adherence to SPs beyond the variables examined in this study. Recent evidence suggests that patient safety climate may influence compliance indirectly through infection prevention climate and individual attitudes toward SPs, underscoring the importance of multilevel organizational and cognitive factors in infection prevention behaviors (21, 25, 26). These findings indicate that adherence to standard precautions is a multifactorial behavior and that future studies should continue to examine additional organizational and workplace-related factors to better explain variations in adherence. Despite these limitations, the present study provides practical insights for policymakers and healthcare managers by identifying key factors associated with adherence to SPs among nurses.

## 5 Conclusion

Adherence to SPs is fundamental to maintaining a safe healthcare environment. This study demonstrated a moderate to high overall level of adherence to SPs, although notable deficiencies remain, particularly in the appropriate disposal of sharps. The findings indicate that greater self-rated familiarity with standard precaution–related knowledge was associated with better adherence, whereas longer nursing experience and higher perceived barriers were associated with lower adherence. These findings highlight the importance of implementing ongoing and tailored educational strategies, addressing workplace barriers, and strengthening institutional support for infection prevention practices to enhance adherence to SPs among nurses.

### 5.1 Implications for nursing practice

This study demonstrates that adherence to SPs is not a uniform behavior, necessitating nuanced infection prevention strategies. The identified gaps in high-risk practices, particularly sharps disposal, suggest that training should shift from generalized guidelines to unit-specific, technical reinforcement. In clinical practice, interventions targeting specific high-risk behaviors and workplace challenges may be more effective than assuming uniform adherence across all components of SPs.

Furthermore, the lower adherence observed among experienced nurses indicates that accumulated clinical tenure does not necessarily guarantee sustained adherence to SPs. As routine practices become habitual over time, protective behaviors may be unintentionally simplified or omitted. Establishing opportunities for periodic reflection, continuing education, and practice review may help experienced nurses maintain consistent adherence to SPs throughout their professional careers.

Additionally, the independent association of perceived barriers underscores the importance of considering nurses’ lived experiences within the clinical environment. Systemic challenges, such as heavy workloads and limited resources, can impede consistent adherence. Regularly eliciting frontline feedback to identify emerging risks may facilitate timely adjustments to clinical support systems and infection prevention practices. Ultimately, aligning infection control protocols with the practical realities of daily nursing work will foster a more resilient and sustainable safety culture.

Although the data were collected during the COVID-19 pandemic, when infection prevention received heightened attention, this provided a valuable context for examining factors associated with nurses’ adherence to standard precautions. Nevertheless, standard precautions remain fundamental to infection prevention and occupational safety and should be consistently implemented before, during, and after a pandemic. Therefore, the principal value of the present study extends beyond the pandemic by identifying factors associated with adherence to standard precautions rather than merely describing adherence during a specific period. These findings provide practical evidence to support infection prevention strategies, continuing education, and long-term efforts to sustain adherence to standard precautions during and beyond the COVID-19 pandemic.

## Data Availability

The raw data supporting the conclusions of this article will be made available by the authors, without undue reservation.
