# Supplementary material for: Factors associated with nurses’ adherence to standard precautions: implications for healthcare-associated infection prevention and occupational safety in Taiwan
Source: Front Public Health. 2026 Aug 10;14:1896298. doi: 10.3389/fpubh.2026.1896298 (PMC13500483; doi:10.3389/fpubh.2026.1896298)
Supplement: Supplementary file 1 [file Table_1.pdf]

Supplementary Table S1.

Exploratory Factor Analysis of the Perceived Barriers Scale and Internal Consistency (N=264).

**Items of perceived barriers (8 items, potential range 0–4)**	*Mean* ± *SD*	Factor loading
Caring for patients with infectious diseases always leaves me exhausted.	3.4 ± 1.2	.850
I feel anxious about contacting patients who are at high risk of infectious diseases.	3.5 ± 1.2	.793
I feel that caring for patients with infectious diseases always affects my sleep.	3.0 ± 1.2	.775
I feel that adhering to standard precautions adds a lot of work stress.	3.0 ± 1.2	.763
Wearing a protective face shield that fogs up hinders my work.	3.4 ± 1.2	.654
I believe being busy at work affects my adherence to standard precautions	3.3 ± 1.4	.653
Wearing an N95 mask makes it difficult for me to breathe.	3.2 ± 1.2	.545
I think that adhering to standard precautions in emergency situations prevents me from immediately addressing patient issues.	3.4 ± 1.3	.499
Explained variance (%)		49.16%
Total Cronbach's alpha		0.844

Note. Items were originally rated on a 5-point Likert scale (1 = strongly disagree, 5 = strongly agree). For factor analysis and internal consistency estimation, item responses were recoded to a 0–4 scale, with higher scores indicating greater perceived barriers. Exploratory factor analysis was performed using principal component analysis with varimax rotation; factor loadings ≥ .40 were considered acceptable. Internal consistency was assessed using Cronbach's alpha.
